# Multi-dimensional patient acuity estimation with longitudinal EHR tokenization and flexible transformer networks

**DOI:** 10.3389/fdgth.2022.1029191

**Published:** 2022-11-09

**Authors:** Benjamin Shickel, Brandon Silva, Tezcan Ozrazgat-Baslanti, Yuanfang Ren, Kia Khezeli, Ziyuan Guan, Patrick J. Tighe, Azra Bihorac, Parisa Rashidi

**Affiliations:** ^1^ Department of Medicine, University of Florida, Gainesville, FL, United States; ^2^ Department of Biomedical Engineering, University of Florida, Gainesville, FL, United States; ^3^ Department of Anesthesiology, University of Florida, Gainesville, FL, United States; ^4^ Intelligent Critical Care Center (IC3), University of Florida, Gainesville, FL, United States

**Keywords:** transformer, deep learning, electronic health records, critical care, patient acuity, clinical decision support

## Abstract

Transformer model architectures have revolutionized the natural language processing (NLP) domain and continue to produce state-of-the-art results in text-based applications. Prior to the emergence of transformers, traditional NLP models such as recurrent and convolutional neural networks demonstrated promising utility for patient-level predictions and health forecasting from longitudinal datasets. However, to our knowledge only few studies have explored transformers for predicting clinical outcomes from electronic health record (EHR) data, and in our estimation, none have adequately derived a health-specific tokenization scheme to fully capture the heterogeneity of EHR systems. In this study, we propose a dynamic method for tokenizing both discrete and continuous patient data, and present a transformer-based classifier utilizing a joint embedding space for integrating disparate temporal patient measurements. We demonstrate the feasibility of our clinical AI framework through multi-task ICU patient acuity estimation, where we simultaneously predict six mortality and readmission outcomes. Our longitudinal EHR tokenization and transformer modeling approaches resulted in more accurate predictions compared with baseline machine learning models, which suggest opportunities for future multimodal data integrations and algorithmic support tools using clinical transformer networks.

## 1. Introduction

Through the course of a typical intensive care unit (ICU) admission, a variety of patient-level data is collected and recorded into electronic health records (EHR) systems. Patient data is diverse, including measurements such as vital signs, laboratory tests, medications, and clinician-judged assessment scores. While primarily used for ad-hoc clinical decision-making and administrative tasks such as billing, patient-centric data can also be used to build automated machine learning systems for assessing overall patient health and predicting recovering or worsening patient trajectories.

Patient mortality risk is often used as a proxy for overall ICU patient acuity, both in traditional illness severity scores like SOFA (1, 2) and more recent machine learning approaches such as DeepSOFA (3). Whether manually calculated or algorithmically computed, nearly all of these systems rely on measurements from a set of handpicked clinical descriptors thought to be most indicative of overall patient health. Given the breadth of data available in modern EHR systems, there is untapped potential for enhanced patient modeling contained in the large amount of unused patient data.

Several recent studies have demonstrated the predictive accuracy and patient modeling capacity of deep learning implementations in healthcare, using models such as recurrent neural networks (RNN) (3–8) and convolutional neural networks (CNN) (9, 10).

Recently, Transformer models (11) have garnered increased attention in the deep learning community due to their state-of-the-art results on a variety of natural language processing (NLP) tasks, particularly when using schemes such as Bidirectional Encoder Representations from Transformers (BERT) (12). There are also more recent advances in analyzing frequency of data in Frequency Enhanced Decomposed Transformer Zhou et al. (13) that exploits the sparseness of time series data.

From a temporal perspective, one advantage the Transformer offers is its parallel processing characteristics. Rather than processing data points sequentially, the Transformer views all available data at once, modeling attention-based relationships between all input time steps. In contrast, models such as RNNs require distinct temporal separation within input sequences, and usually demand a regular sample interval between adjacent time steps. As clinical EHR data is recorded at highly irregular frequency and is often missing measurements, a large amount of data preprocessing is typically required in the form of temporal resampling to a fixed frequency, and an imputation scheme to replace missing values. Furthermore, given that several EHR measurements are often recorded at the same timestamp, typical machine learning workflows aggregate temporally adjacent measurements into mean values contained in resampled time step windows, or perform random shuffling procedures before training models. Given its parallel and fundamentally temporally agnostic attributes, the Transformer is capable of distinctly processing all available measurements, even those occurring at the same timestamp. Additionally, the Transformer is able to process whichever data happens to be available, reducing the need for potentially bias-prone techniques to account for data missingness.

In this study, we showcase the feasibility of a highly flexible Transformer-based patient acuity prediction framework in the critical care setting. Our contributions can be summarized by the following:

• Our flexible system design incorporates a diverse set of EHR input data that does not require *a priori* identification of clinically relevant input variables, and can work with any data contained in EHR platforms.
• In contrast to recent Transformer approaches that either use discrete medical concepts (14–16) or continuous measurements from a handpicked set of features (17), we introduce a data embedding scheme that jointly captures both concept and corresponding measurement values of a wide variety of disjoint clinical descriptors.
• In our novel embedding module, we introduce a mechanism for combining both absolute and relative temporality as an improvement over traditional positional encoding.
• We present an input data scheme with minimal preprocessing, obfuscating the need for potentially biased temporal resampling or missing value imputation common in many other sequential machine learning approaches.
• We expand BERT’s [CLS] token for classification into several distinct tokens for predicting multiple-horizon patient mortality and ICU readmission in a novel multi-task learning environment.
• Rather than typical concatenation with sequential representation, we incorporate static patient information in a novel way using a global self-attention token so that every sequential time step is compared with the static pre-ICU representation.
• We show that the Longformer (18) can be applied to long EHR patient data sequences to minimize required computation while retaining superior performance.

## 2. Methods

### 2.1. Cohort

The University of Florida Integrated Data Repository was used as an honest broker to build a single-center longitudinal dataset from a cohort of adult patients admitted to intensive care units at University of Florida Health between January 1st, 2012 and September 22nd, 2019. Our project was approved by the Institutional Review Board of the University of Florida and the University of Florida Privacy Office (IRB201901123). Full cohort statistics is described in Table 1.

**Table 1:** Summary statistics for experimental ICU cohorts.

	Development cohort (n = 60, 516)	Validation cohort (n = 12, 674)
Patients, n	41,881	10,315
Hospital encounters, n	57,168	12,127
Age, years, median (25th, 75th)	61.0 (49.0, 71.0)	62.0 (49.0, 73.0)
Female, n (%)	27,380 (45.2)	5,616 (44.3)
Body mass index, median (25th, 75th)	26.9 (23.0, 32.0)	27.3 (23.3, 32.2)
Hospital length of stay, days, median (25th, 75th)	6.7 (3.6, 12.1)	6.4 (3.3, 11.5)
ICU length of stay, days, median (25th, 75th)	2.8 (1.5, 5.1)	2.9 (1.6, 5.5)
Time to hospital discharge, days, median (25th, 75th)	1.9 (0.0, 4.8)	1.1 (0.0, 4.1)
Hispanic, n (%)	2,130 (3.5)	539 (4.3)
Non-English speaking, n (%)	1,092 (1.8)	233 (1.8)
Marital status, n (%)		
Married	26,084 (43.1)	5,457 (43.1)
Single	21,844 (36.1)	4,931 (38.9)
Divorced	11,905 (19.7)	2,142 (16.9)
Smoking status, n (%)		
Never	20,180 (33.3)	4,653 (36.7)
Former	19,378 (32.0)	4,167 (32.9)
Current	12,094 (20.0)	2,326 (18.4)
Insurance status, n (%)		
Medicare	31,447 (52.0)	6,543 (51.6)
Private	13,115 (21.7)	2,912 (23.0)
Medicaid	10,208 (16.9)	1,999 (15.8)
Uninsured	5,746 (9.5)	1,220 (9.6)
Comorbidities, n (%)		
Charlson comorbidity index, median (25th, 75th)	2.0 (0.0, 4.0)	2.0 (0.0, 4.0)
Myocardial infarction	7,537 (12.5)	1,985 (15.7)
Congestive heart failure	14,897 (24.6)	3,380 (26.7)
Peripheral vascular disease	10,005 (16.5)	2,185 (17.2)
Cerebrovascular disease	8,981 (14.8)	1,720 (13.6)
Chronic pulmonary disease	17,938 (29.6)	3,473 (27.4)
Metastatic carcinoma	3,377 (5.6)	812 (6.4)
Cancer	8202 (13.6)	1,808 (14.3)
Mild liver disease	4,745 (7.8)	960 (7.6)
Moderate/severe liver disease	1,856 (3.1)	374 (3.0)
Diabetes without complications	14,137 (23.4)	2,395 (18.9)
Diabetes with complications	5,052 (8.3)	1,736 (13.7)
AIDS	442 (0.7)	53 (0.4)
Dementia	1,692 (2.8)	559 (4.4)
Paraplegia/hemiplegia	3,465 (5.7)	769 (6.1)
Peptic ulcer disease	1,110 (1.8)	187 (1.5)
Renal disease	11,878 (19.6)	2,493 (19.7)
Rheumatologic disease	1,794 (3.0)	342 (2.7)
Neighborhood characteristics, median (25th, 75th)		
Total population, $n\times 10^{3}$	17.0 (10.6, 26.4)	17.6 (10.6, 26.7)
Distance to hospital, km	39.3 (17.9, 69.1)	42.4 (20.2, 76.5)
Median income, dollars $\times 10^{3}$	40.1 (33.8, 46.7)	40.1 (35.1, 47.4)
Poverty rate, %	19.6 (14.0, 27.7)	19.3 (13.7, 26.7)
Rural area, n	22543 (37.3)	4691 (37.0)
Clinical outcomes, n (%)		
ICU readmission before hospital discharge	3,583 (5.9)	613 (4.8)
Inpatient mortality	5,813 (9.6)	1,131 (8.9)
7-day mortality	5,237 (8.7)	1,022 (8.1)
30-day mortality	7,056 (11.7)	1,380 (10.9)
90-day mortality	9,197 (15.2)	1,785 (14.1)
1-year mortality	12,991 (21.5)	2,288 (18.1)

We excluded ICU stays lasting less than 1 h (to reduce EHR data artifacts and provide predictive models with adequate patient data) or more than 10 days, to limit outliers based on tokenized sequence length and following several existing studies using ICU encounters for predictive modeling (19). Excluding patients based on length of stay resulted in roughly 95% of the original ICU cohort. Our final cohort consisted of 73,190 distinct ICU stays from 69,295 hospital admissions and 52,196 unique patients. The median length of stay in the ICU was 2.7 days.

We divided our total cohort of ICU stays into a development cohort of 60,516 ICU stays (80%) for training our models, and a validation cohort of 12,674 ICU stays (20%) for evaluating their predictive performance. 10% of the development set was used for within-training validation and early stopping. The cohort was split chronologically, where the earliest 80% of ICU stays was used for training, and the most recent 20% used for evaluation. To ensure the same patient did not appear in both development and validation sets, all ICU stays of patients with multiple admissions spanning the cohort threshold were grouped into the development cohort.

### 2.2. Data

We extracted patient data from several EHR data sources: sociodemographics and information available upon hospital admission, summarized patient history, vital signs, laboratory tests, medication administrations, and numerical assessments from a variety of bedside scoring systems. We did not target or manually select any specific ICU variables, instead using all such data contained in our EHR system. A full list of variables used in our experiments is shown in Table 2.

**Table 2:** Summary of variables used in Transformer experiments.

Variable	Type
*Patient demographics*	
Age	Static
Sex	Static
Ethnicity	Static
Race	Static
Language	Static
Marital status	Static
Smoking status	Static
Insurance provider	Static
*Patient residential information*	
Total population	Static
Distance from hospital	Static
Rural/Urban	Static
Median income	Static
Proportion black	Static
Proportion hispanic	Static
Percent below poverty line	Static
*Patient admission information*	
Height	Static
Weight	Static
Body mass index	Static
17 comorbidities present at Admission	Static
Charlson comorbidity index	Static
Presence of chronic kidney disease	Static
Admission type	Static
*Patient history: medications* ^a^	
ACE inhibitors	Static
Aminoglycosides	Static
Antiemetics	Static
Aspirin	Static
Beta blockers	Static
Bicarbonates	Static
Corticosteroids	Static
Diuretics	Static
NSAIDS	Static
Vasopressors/Inotropes	Static
Statins	Static
Vancomycin	Static
Nephrotoxic drugs	Static
*Patient history: laboratory test results* ^b^	
Serum hemoglobin	Static
Urine hemoglobin	Static
Serum glucose	Static
Urine glucose	Static
Urine red blood cells	Static
Urine protein	Static
Serum urea nitrogen	Static
Serum creatinine	Static
Serum calcium	Static
Serum sodium	Static
Serum potassium	Static
Serum chloride	Static
Serum carbon dioxide	Static
White blood cells	Static
Mean corpuscular volume	Static
Mean corpuscular hemoglobin	Static
Hemoglobin concentration	Static
Red blood cell distribution	Static
Platelets	Static
Mean platelet volume	Static
Serum anion gap	Static
Blood pH	Static
Serum oxygen	Static
Bicarbonate	Static
Base deficit	Static
Oxygen saturation	Static
Band count	Static
Bilirubin	Static
C-reactive protein	Static
Erythrocyte sedimentation rate	Static
Lactate	Static
Troponin T/I	Static
Albumin	Static
Alaninen	Static
Asparaten	Static
**ICU vital signs**	
Systolic blood pressure^c^	Temporal
Diastolic blood pressure^c^	Temporal
Mean arterial pressure^c^	Temporal
Heart rate	Temporal
Respiratory rate	Temporal
Oxygen flow rate	Temporal
Fraction of inspired oxygen (FIO2)	Temporal
Oxygen saturation (SPO2)	Temporal
End-tidal carbon dioxide (ETCO2)	Temporal
Minimum alveolar concentration (MAC)	Temporal
Positive end-expiratory pressure (PEEP)	Temporal
Peak inspiratory pressure (PIP)	Temporal
Tidal volume	Temporal
Temperature	Temporal
*ICU Assessment Scores* ^d^	
ASA physical status classification	Temporal
Braden scale	Temporal
Confusion assessment method (CAM)	Temporal
Modified early warning score (MEWS)	Temporal
Morse fall scale (MFS)	Temporal
Pain score	Temporal
Richmond agitation-sedation scale (RASS)	Temporal
Sequential organ failure assessment (SOFA)	Temporal
*ICU laboratory tests* ^e^	
106 distinct lab tests present in EHR system	Temporal
*ICU medications* ^e^	
345 distinct medications present in EHR system	Temporal

^a^ Extracted features included total counts of administered medications up to one year prior to hospital admission.

^b^ Extracted features included total counts of recorded laboratory test results and minimum, maximum, mean, and standard deviation of measurement values up to one year prior to hospital admission. Both serum and urine-based tests extracted separately when available.

^c^ Invasive and non-invasive readings for systolic blood pressure, diastolic blood pressure, and mean arterial pressure were treated as distinct event tokens.

^d^ For assessment scores with multiple sub-components, each component was treated as a distinct timestamped measurement, resulting in 30 such assessment measurements.

^e^ We retained distinct laboratory tests and medications that were administered in at least 1% of the training cohort of ICU stays.

**Static data**: For each ICU stay, we extracted a set of non-sequential clinical descriptors pertaining to patient characteristics, admission information, and a summarized patient history from the previous year. Patient-level features included several demographic indicators, comorbidities, admission type, and neighborhood characteristics derived from the patient’s zip code. Patient history consisted of a variety of medications and laboratory test results up to one year prior to hospital admission (Table 2). Historical patient measurement features were derived from a set of statistical summaries for each descriptor (minimum, maximum, mean, standard deviation).

**Temporal data**: For each ICU stay, we extracted all available vital signs, laboratory tests, medication administrations, and bedside assessment scores recorded in our EHR system while the patient was in the ICU (Table 2). We refer to each extracted measurement as a clinical event. Each event was represented as a vector containing the name of the measurement (e.g. “noninvasive systolic blood pressure”), the elapsed time from ICU admission, the current measured value, and eight cumulative value-derived features corresponding to prior measurements of the same variable earlier in the ICU stay (mean, median, count, minimum, maximum, standard deviation, first value, elapsed time since most recent measurement). For bedside assessment scores with multiple sub-components, we treated each sub-component as a distinct measurement. Invasive and noninvasive measurements were treated as distinct tokens. We excluded ICU stays with sequence lengths longer than 12,000 tokens, and the resulting mean sequence length in our cohorts was 1,996.

**Data processing**: Categorical features present in the pre-ICU static data were converted to one-hot vectors and concatenated with the remaining numerical features. Missing static features were imputed with training cohort medians, but no such imputation was required for the tokenized temporal ICU data. Binary indicator masks were computed and concatenated with static features to capture patterns of missingness.

Static features were standardized to zero mean and unit variance based on values from the training set. For each variable name in the temporal ICU data, corresponding continuous measurement value features were individually standardized in the same manner. ICU measurement timestamps were converted to number of elapsed hours from ICU admission, and were similarly standardized based on training cohort values.

ICU measurement names were converted to unique integer identifiers in a similar manner to standard tokenization mapping procedures in NLP applications. Each temporal clinical event was also associated with an integer position index. While similar to the positional formulations in NLP applications, we introduce one key distinction that is more suitable for Transformers based on EHR data: we do not enforce the restriction that positional indices are unique, and if two clinical events occurred at the same EHR timestamp, they are associated with the same sequential position index.

Each temporal measurement token consisted of integer positional identifier, integer variable identifier, continuous elapsed time from ICU admission, and eight continuous features extracted from current and prior measurement values.

Following data extraction and processing, each ICU stay was associated with two sets of data: (1) a single vector $x_{s}\in R^{718\times 1}$ of 718 static pre-ICU features, and (2) a matrix of T temporal ICU measurements $x_{t}\in R^{T\times 12}$ including token position and identifier. Across our entire population, the temporal ICU measurements included 19 unique vital signs, 106 unique laboratory tests, 345 unique medication administrations, and 29 bedside assessment score components; however, each ICU stay only included a subset of such total variables, and its corresponding temporal sequence only included what was measured during the corresponding ICU stay. One of the benefits of our proposed EHR embedding framework is the lack of resampling, propagation, imputation, or other such temporal preprocessing typically performed in related sequential modeling tasks.

### 2.3. Clinical outcomes

For each ICU stay, we sought to predict six clinical outcomes related to patient illness severity: ICU readmission within the same hospital encounter, inpatient mortality, 7-day mortality, 30-day mortality, 90-day mortality, and 1-year mortality. Our model is formulated as a multi-task design, and simultaneously estimates risk for all six clinical prediction targets.

### 2.4. Model architecture

**Figure 1:**
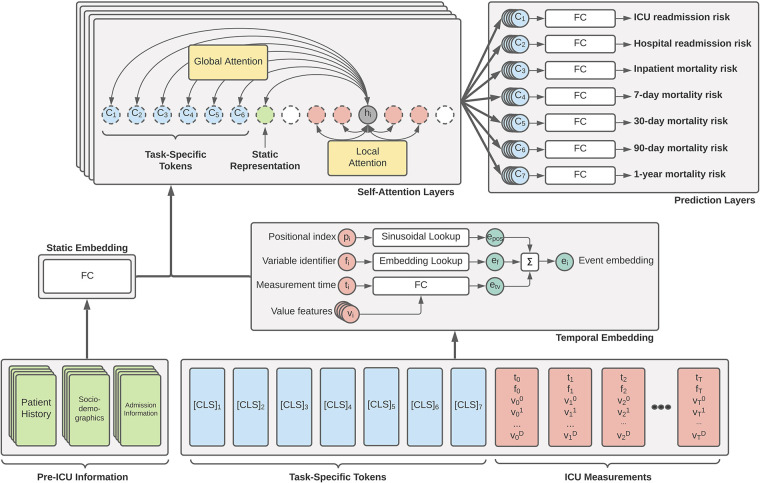
Overview of our proposed generalized EHR Longformer network for simultaneously predicting multiple patient outcomes in the ICU. Pre-ICU information includes summarized history of patient medications and laboratory tests, sociodemographic indicators, and features relating to hospital admission. Temporal ICU measurements take the flexible form of tuples: (*p*, non-unique positional index of clinical event based on timestamp; *t*, elapsed time from ICU admission, *f*, unique measurement identifier integer; $\vec{v}$, set of continuous features derived from measured values). Task-specific [CLS] tokens are assigned t = time of prediction and $\vec{v}=0$. Tokens are individually embedded and passed through a stack of Longformer layers with sliding self-attention windows. Global attention is applied to static feature representation and prediction tokens. The concatenation of each layer’s [CLS] representations are used for a given task to predict the desired mortality risk. Not shown: Transformer feedforward network and nonlinear activations. FC: fully-connected layers.

The primary driver behind our ICU patient acuity estimation model is the transformer encoder (11). Our modified model utilizes the global and sliding window mechanism introduced by the Longformer (18) along with special classification tokens from BERT (12). Figure 1 shows a high-level overview of our Transformer architecture. Our longitudinal tokenization pipeline and Transformer modeling architecture code will be available upon request for interested researchers.

**Novel embedding**: In typical Transformer implementations, one-dimensional input sequences consist of integer-identified tokens (such as textual tokens or discrete clinical concepts) that are embedded using a lookup table, after which a positional encoding vector is added to inject local temporality. For existing applications of Transformers with EHR data, the values of a given measurement are not factored into its representation.

Our embedding scheme introduces three novelties that offer improvements for clinical prediction tasks. First, positional indices are derived from EHR record times and are not unique (see Section 2.2), allowing for multiple tokens to share the same positional index and resulting positional encoding. Rather than enforce an arbitrary sequence order or implement a random shuffling procedure for simultaneous tokenized events, this modification is more flexible with respect to clinical workflows.

Second, in addition to novel framing of relative and local temporal relationships through positional encoding modifications, each clinical event token also explicitly includes absolute temporality in the form of a feature indicating the elapsed hours from ICU admission. We hypothesized that the injection of both relative and absolute temporality would allow the Transformer to better model patient trajectories.

Finally, each clinical event in our tokenized input sequences consists of several continuous measurement values in addition to the discrete token identifiers (see Section 2.2). To our knowledge, no other work integrates both discrete and continuous data in this manner, with the majority of recent research opting for discrete medical codes only (Section 4.2). We augment discrete variable tokens with continuous measurement values into our embedding to better capture recovery or worsening trends as a patient progresses through an ICU stay.

Our embedding module consists of (1) a traditional lookup table used for measurement name identifier, (2) a sinusoidal positional embedding table, and (3) a single fully-connected layer for embedding absolute time and value-derived features. The final sequence embedding is the summation of three embedded vectors: (1) the embedding of absolute time with corresponding cumulative values, (2) the measurement token identifier embedding, and (3) a traditional sinusoidal positional encoding. In our implementation, the sinusoidal positional encoding is based on the position of unique measurement times in the input sequence: for an example sequence of measurement hours [0.1,0.2,0.2,0.3,0.3], the positional indices are computed as [0,1,1,2,2].

**Novel multi-task global tokens**: In the original BERT implementation, a single special [CLS] token is prepended to input sequences that is meant to capture a global representation of the entire sequence. We extend this notion by prepending each sequence with 6 such special tokens: one for each of our clinical outcomes. As each token in our data scheme consists of a (time, name, values) 12-tuple, we set time of each [CLS] token equal to the total ICU length of stay and all values equal to zero. The special token identifiers are embedded in a similar fashion to other ICU measurement tokens. In our experiments, we include an additional prediction target for long-term hospital readmission that is used for regularization, but not included in our patient acuity estimation. In the Longformer implementation in our encoder, we set each of the multi-task tokens to compute global attention, so that self-attentions are computed among all sequence elements for each clinical outcome token.

**Novel inclusion of static patient data**: In many sequential models for clinical prediction, a final encounter representation is obtained by concatenating the pre-sequence static patient representation with the sequential representation. In our work, we prepend each ICU sequence with the representation obtained from passing the static patient information vector through a fully-connected network. We assign this static token as global, so that every time step computes attention with the static data. We hypothesized that this more fine-grained injection of patient information at every time step would improve the capacity of our model to learn important and more personalized patient trajectory patterns.

**Model details**: Our final model consisted of an embedding layer, followed by 8 Longformer layers, and a separate linear prediction layer for each of our 6 clinical outcomes. For making a task-specific prediction, the task-specific linear layer uses the concatenation of representations corresponding to its special [CLS] token at each of the 8 layers. In our initial Longformer implementation, we used a hidden size of 128, a feedforward size of 512, 8 attention heads, a sliding window of size 128, dropout of 0.1, and a batch size of 21. Hyperparameters were chosen with respect to hardware constraints; hyperparameter optimization will be a focus of future work.

**Experiment details**: Models were trained using a development set of 60,516 ICU stays corresponding to 80% of our total ICU cohort. 10% of this development set was used for early stopping based on the mean AUROC among all six clinical outcomes and a patience of four epochs. All experiments were conducted on a local Linux server equipped with two i7-7820X 3.6 GHz CPUs, 3 NVIDIA GeForce RTX 2080Ti GPUs, 512GB SSD storage, and 128GB RAM. Models were designed and run using the PyTorch and Hugging Face Python libraries.

In this feasibility study, we compared performance against six other ICU prediction models:

• Longformer using tokenized data sequences with only discrete code identifiers. In this variant of our proposed framework, we do not include the continuous measurement values in the representation of each event token.
• Recurrent neural network (RNN) with gated recurrent units (GRU) using continuous multivariate time series inputs. In this experiment, the flexibility of our tokenization scheme is removed, and more traditional “tabularized” input data sequences were constructed where each variable is assigned a distinct column. Sequences were constructed with continuous current values and resampled to 1-hour frequency to align with common practice found in literature. Multi-task predictions were drawn from the final hidden state of the GRU encoder. Static patient information was concatenated with the sequence representation and fed through fully-connected layers before classification.
• GRU with attention mechanism. This variant is identical to the above, but with the addition of a simple attention mechanism over the hidden states of the GRU. States are weighted by alignment scores and summed to yield a final attention-based sequential representation.
• Tokenized GRU with attention. In this final experimental setting, we used the same novel EHR embedding and tokenization approach as with our Transformer model architecture (see Section 2.2), but instead use a GRU with attention mechanism in place of the Transformer model.
• CatBoost (20) gradient boosting algorithm. The algorithm employs gradient boosting on decision trees for both regression and classification tasks. Gradient boosting algorithms have shown benefits over random forests and require comparatively less hyperparameter tuning for optimal performance. For this experiment, the embedding layers are removed and the CatBoost model is trained on samples containing both the pre-ICU information and concatenated ICU measurements.
• XGBoost (21) gradient boosting algorithm. This experiment and associated data processing is identical to CatBoost, except an XGBoost model is used for prediction.

## 3. Results

At present time, the primary aim of our novel mortality prediction model is not to show state-of-the-art improvements in model accuracy; rather, we present this work as a feasibility study for future research. We believe our novel modifications of existing Transformer architectures for use in clinical EHR applications will result in highly flexible and more personalized patient representations and predictions across a variety of clinical tasks.

In this first iteration of our experiments, we did not perform any hyperparameter optimization, instead choosing sensible settings that both highlight the novel aspects of the architecture and work with our hardware constraints. In passing, we note that often parameter tuning is an essential component of enhancing performance, and future iterations of this work will focus on optimizing crucial parameters such as learning rate, dropout, number of self-attention heads, number of self-attention layers, hidden dimension, and size of the sliding self-attention window.

Our results are shown in Table 3. Our Transformer architecture with novel EHR embedding and tokenization scheme yielded slightly superior mean AUROC (0.929) across all six clinical prediction tasks, with individual task AUROC ranging from 0.843 (ICU readmission) to 0.983 (7-day mortality). The Transformer using tokenized embeddings that omit continuous measurement values resulted in the lowest mean AUROC (0.773) and worst performance across most of the clinical outcomes, ranging from 0.512 (ICU readmission) to 0.900 (7-day mortality). It outperformed the XGBoost model for inpatient and 7-day mortality.

**Table 3:** Multi-task prediction results expressed as area under the receiver operating characteristic curve (AUROC).

Model	Data	Mean	Readmission	Mortality				
			ICU	Inpatient	7-Day	30-Day	90-Day	1-Year
Transformer	Tokenized events (discrete only)	0.773	0.512	0.889	0.900	0.831	0.777	0.727
Transformer	Tokenized events + continuous measurement values	**0.929**	**0.843**	**0.978**	**0.983**	0.953	0.923	**0.892**
GRU	Resampled multivariate time series	0.900	0.750	0.960	0.972	0.938	0.907	0.872
GRU with attention	Resampled multivariate time series	0.909	0.770	0.965	0.975	0.946	0.914	0.882
GRU with attention	Tokenized events + continuous measurement values	0.927	0.831	0.977	0.982	**0.954**	**0.925**	0.891
CatBoost	Tokenized events + continuous measurement values	0.863	0.759	0.901	0.915	0.890	0.868	0.847
XGBoost	Tokenized events + continuous measurement values	0.836	0.762	0.867	0.878	0.859	0.833	0.817

In terms of GRU baseline models, the traditional model and data processing scheme resulted in the lowest baseline accuracy, with mean AUROC of 0.900 and task AUROC ranging from 0.750 (ICU readmission) to 0.972 (7-day mortality). The augmentation of this model and data scheme with traditional attention mechanism improved the performance to a mean AUROC of 0.909.

The best GRU baseline model used our novel EHR embedding, tokenization, and representation pipeline. This model yielded a mean AUROC of 0.927 with individual task AUROC ranging from 0.831 to 0.982. It performed best for predicting 30-day mortality and 90-day mortality, although the relative difference compared with the transformer is minimal. For the gradient boosting algorithms, CatBoost outperformed XGBoost across all outcomes (mean AUROC: 0.863 vs. 0.836) except for predicting ICU readmission (AUROC: 0.759 vs. 0.762). The CatBoost model performed similarly to the baseline GRU model for all other outcomes. The tree-based models were predominantly outperformed by GRU models with attention.

Across all models and data representation schema, ICU readmission proved the most difficult task. Among the multiple prediction horizons for patient mortality, models were best able to predict 7-day mortality, followed by inpatient mortality, 30-day mortality, 90-day mortality, and 1-year mortality.

## 4. Discussion

### 4.1. Principal findings

This work presents a novel ICU acuity estimation model inspired by recent breakthroughs in Transformer architectures. Our proposed model framework incorporates several novel modifications to the existing Transformer architecture that make it more suitable for processing EHR data of varying modalities. Through initial feasibility experiments, our model was on par with, or outperformed, common variants of RNN baselines, and we feel our approach holds promise for incorporating additional EHR-related outcome prediction tasks and additional sources of EHR input data.

One of the advantages of our work is that input elements are treated as distinct. For example, if heart rate, respiratory rate, and SPO2 were recorded at the same timestamp in an EHR system, our framework operates on these individual elements, rather than combining them into a single aggregated time step as in similar RNN or CNN-based work. From an interpretability standpoint, combined with the inherent self-attention mechanisms of the Transformer, isolation of inputs allows for improved clarity with respect to important or contributing clinical factors. While one area of recent sequential interpretability research involves multivariate attribution for aggregated time steps (5, 22), Transformer-based approaches such as ours obfuscate the need for multivariate attribution, as attentional alignment scores are assigned to individual measurements. This property highlights the potential for EHR Transformers to shed increased transparency and understanding for clinical prediction tasks built upon complex human physiology.

Furthermore, while many sequential applications of deep learning to EHR (including recent implementations of Transformer techniques) make use only of discrete clinical concepts, our proposed framework extends the representational capacity by integrating continuous measurement values alongside these discrete codes and events. The inclusion of continuous measurement values represents an important step forward, as the measured result of a clinical test or assessment can provide crucial information alongside a simple presence indicator that can help complex models develop a better understanding of patient state and overall health trajectory.

Given the flexible nature of our Transformer framework, each patient input sequence only contains the measurements that were made during the ICU encounter. The advantages for EHR applications are twofold. First, in traditional RNN or CNN-based work, the distance between time steps is assumed to be fixed, and this is typically achieved by resampling input sequences to a fixed frequency by aggregating measurements within resampled windows, and propagating or imputing values into windows without present values. Such a scheme has the potential for introducing bias, and when using our novel EHR embedding paradigm and Transformer-based modeling approach, the problem of missing values is made redundant given the explicit integration of both absolute and relative temporality for each irregularly measured clinical event. Additionally, in typical deep sequential applications using EHR data, the number of input features at each time step must be constant. This is achieved by an *a priori* identification and extraction of a subset of clinical descriptors thought to be relevant indicators for a given prediction task. As we have shown, when using a Transformer-based approach with our flexible tokenization scheme, any and all EHR measurements can be easily incorporated into the prediction framework, even when some types do not exist for a given patient or ICU encounter, and do not necessitate bias-prone imputation techniques.

While the Transformer offers several benefits over existing sequential deep learning models such as the RNN, it is not without drawbacks. Because the self-attention mechanism is highly parallelizable and does not require step-wise iterative processing of a sequence (unlike the RNN), there is a tradeoff between faster computation and a much larger memory footprint (complexity $O(n^{2})$ without scope modifications). As such, Transformers may be infeasible to implement in training environments with limited computational resources.

In our approach, we introduced a novel method for incorporating static, pre-sequential patient information and patient history into the overall prediction model. Typically, such static information is concatenated with a final sequential representation before making a prediction. We instead include static information as a distinct token in the input sequence, and assign global attention using the Longformer self-attention patterns. In effect, static patient-level information is injected into the self-attention representation of every ICU measurement, allowing more fine-grained and personalized incorporation of changes in overall patient health trajectories.

Another novel contribution we feel can be applied to even non-EHR tasks is the expansion of the special BERT classification token into a separate token per classification target in a multi-task prediction setting. Given the global self-attention patterns between all task tokens and every sequential input element, such a scheme allows the model to develop task-specific data representations that can additionally learn from each other.

As with other retrospective machine learning models for predicting patient outcomes from longitudinal data, our transformer framework offers the potential for augmenting clinical decision-making with dynamic data-driven risk estimations that can be used to help forecast patient trajectory and guide treatment and care strategies. Intended not to mandate particular course of action, tools such as ours can complement existing standards of care and provide clinicians with additional support.

### 4.2. Related work

#### 4.2.1. Transformer models

First introduced by Vaswani et al. (11) for machine translation tasks, the Transformer is a deep learning architecture built upon layers of self-attention mechanisms. The Transformer views attention as a function of keys K, queries Q, and values V. In the work of Vaswani et al. (11), all three elements came from the same input sequence, and is why their style of attention is referred to as self-attention. In a similar manner to previously described works, compatibility between a key and query is used to weight the value, and in the case of self-attention, each element of an input sequence is represented as a contextual sum of the alignment between itself and every other element. Similar to the memory networks of Sukhbaatar and Szlam (23), the Transformer also involves the addition of a positional encoding vector to preserve relative order information between input tokens.

An end-to-end Transformer architecture typically includes both an encoder and decoder component. While critical for many NLP tasks such as machine translation, our architecture utilizes only the Transformer encoder, which encodes input sequences into hidden representations that are subsequently used for predicting patient mortality.

A comprehensive overview of the Transformer and BERT is beyond the scope of this section; we refer interested readers to Vaswani et al. (11) and Devlin et al. (12), respectively.

Briefly, the first stage of a Transformer encoder typically includes an embedding component, where each input sequence element is converted to a hidden representation that is fed into the remainder of the model. In its original NLP-centered design where inputs are sequences of textual tokens, a traditional embedding lookup table is employed to convert such tokens into continuous representations. Unlike similar sequential models like RNNs or CNNs, the Transformer is fundamentally temporally agnostic and processes all tokens simultaneously rather than sequentially. As such, the Transformer embedding module must inject some notion of temporality into its element embeddings. In typical Transformer implementations, this takes the form of a positional encoding vector, where the position of each element is embedded by sinusoidal lookup tables, which is subsequently added to the token embeddings. The primary aim of such positional embeddings is to allow the model to understand local temporality between nearby sequence elements.

At each layer of a Transformer encoder, a representation of every input sequence element is formed by summing self-attention compatibility scores between the element and every other element in the sequence. Typical with other deep learning architectures, as more layers are added to the encoder, the representations become more abstract.

The recent NLP method BERT (12) is based on Transformers, and at present time represent state of the art in a variety of natural language processing tasks. In addition to its novel pretraining scheme, BERT also prepends input sequences with a special [CLS] token before a sequence is passed through the model. The goal of this special token is to capture the combined representation of the entire sequence, and for classification tasks is used for making predictions.

Transformers are also being used in computer vision as well, with great success. For example, videos especially benefit from Transformers which can learn the temporal and spatial features of vision data. They have shown to before the same or better for vision tasks, while also reducing vision-specific induction bias Han et al. (24). For video data, they can be used for trajectory tracking of objects like balls Patrick et al. (25) using attention on objects in images, as well as approximate self attention to reduce quadratic dependency.

While the Transformer is in one sense more efficient than its sequential counterparts due to its ability to parallelize computations at each layer, one of the main drawbacks is its required memory consumption. Since each input element of a sequence of length n must be compared with every other input element in the sequence, typical Transformer implementations require memory on the order of $O(n^{2})$. While acceptable for relatively short sequences, the memory consumption quickly becomes problematic for very long sequences. Decreasing the memory requirement of Transformers is an area of ongoing research.

One potential solution was proposed by Beltagy et al. (18) in their Longformer architecture. Rather than computing full $n^{2}$ self-attentions, they propose a sliding self-attention window of specified width, where each input sequence element is compared only with neighboring sequence elements within the window. They extend this to include user-specified global attention patterns (such as on the special [CLS] tokens for classification) that are always compared with every element in the sequence. Through several NLP experiments, they demonstrate the promising ability of the Longformer to approximate results from a full Transformer model.

#### 4.2.2. Transformers in healthcare

Given the similarity between textual sequences and temporal patient data contained in longitudinal EHR records, several works have begun exploring the efficacy of Transformers and modifications of BERT for clinical applications using electronic health records. In terms of patient data modalities, existing implementations of Transformers in a clinical setting tend to fall under three primary categories:

Perhaps the most aligned with the original BERT implementation, several studies adapt and modify BERT for constructing language models from unstructured text contained in clinical notes. The ClinicalBERT framework of Huang et al. (26) used a BERT model for learning continuous representations of clinical notes for predicting 30-day hospital readmission. Zhang et al. (27) pretrained a BERT model on clinical notes to characterize inherent bias and fairness in clinical language models.

Song et al. (17)’s SAnD architecture developed Transformer models for several clinical prediction tasks using continuous multivariate clinical time series.

The majority of existing EHR Transformer research has focused on temporal sequences of discrete EHR billing codes. Li et al. (16)’s BEHRT framework modified the BERT paradigm for predicting future disease from diagnosis codes. Med-BERT (15) demonstrated the performance advantages of a contextualized clinical pretraining scheme in conjunction with a BERT modification. RAPT (28) used a modified Transformer pretraining scheme to overcome several challenges with sparse EHR data. SETOR (29) utilized neural ordinary differential equations with medical ontologies to construct a Transformer model for predicting future diagnoses. RareBERT (30) extends Med-BERT for diagnosis of rare diseases. Meng et al. (31) used Transformers for predicting depression from EHR. Hi-BEHRT (16) extends BEHRT using a hierarchical design to expand the receptive field to capture longer patient sequences. Choi et al. (32) and Shang et al. (33)’s G-BERT architecture capitalize on the inherent ontological EHR structure.

In contrast to the isolated data modalities implemented in existing EHR Transformers, the novel embedding scheme utilized in our models combines both discrete and continuous patient data to generate a comprehensive representation of distinct clinical events and measurements.

### 4.5. Limitations

This feasibility study has several limitations and is intended as a methodological guiding framework for future multimodal and multi-task EHR Transformer research. Our retrospective dataset is limited to patients from a single-center cohort. Future work will evaluate performance in external validation cohorts such as MIMIC-IV (34). We also present results with parameters that maximize our limited hardware capacity; future work will focus on several hyperparameter tuning and model selection procedures. The baseline models we present for comparison are drawn from simplified implementations found in clinical deep learning research, and more recent approaches may offer enhanced predictive performance. From the results in Table 3, one might conclude that our EHR embedding procedure had a larger impact than use of the Transformer architecture, given the competitive AUROC of the attentional GRU baseline when implementing our tokenization pipeline for estimating risk of patient mortality. Future work will focus on disentangling the relative impacts of both model and data representation designs.

### 4.6. Conclusions and next steps

We feel there is still great potential for exploring additional benefits of our approach with diverse EHR data for a variety of clinical modeling and prediction tasks, especially in the realm of clinical interpretability. Given our promising pilot study results, future versions of this work will perform hyperparameter optimization with a focus on maximizing predictive accuracy. Additionally, since transformers are fundamentally composed of attention mechanisms, they can be analyzed with respect to particular outcomes, time points, or variables of interest to highlight important contributing factors to overall risk estimation. Future research will emphasize analyzing self-attention distributions between input variables and clinical outcomes to further the clinical explainability and enhance the clinical trust of Transformers in healthcare. We believe there is great potential for multimodal patient monitoring using flexible EHR frameworks such as ours. Future research will also focus on augmenting our multi-modal datasets with additional clinical data modalities such as clinical text and images, and pre-training our Transformer architectures with self-supervised prediction schemes across a variety of input data and clinical outcomes.

## Data Availability

The data analyzed in this study is subject to the following licenses/restrictions: UFHealth cohort data are available from the University of Florida Institutional Data Access/Ethics Committee for researchers who meet the criteria for access to confidential data and may require additional IRB approval. Requests to access these datasets should be directed to https://idr.ufhealth.org.

## References

[1] Vincent JL Moreno R Takala J Willatts S De Mendonca A Bruining H The SOFA (Sepsis-related Organ Failure Assessment) score to describe organ dysfunction/failure. On behalf of the working group on sepsis-related problems of the european society of intensive care medicine. Intensive Care Med (1996) 22:707–10. 10.1007/BF01709751 8844239

[2] Vincent JL de Mendonca A Cantraine F Moreno R Takala J Suter PM, Use of the SOFA score to assess the incidence of organ dysfunction/failure in intensive care units. Crit Care Med (1998) 26:1793–800. 10.1097/00003246-199811000-00016 9824069

[3] Shickel B Loftus TJ Adhikari L Ozrazgat-Baslanti T Bihorac A Rashidi P. DeepSOFA: a continuous acuity score for critically ill patients using clinically interpretable deep learning. Sci Rep (2019) 9:1879. 10.1038/s41598-019-38491-0 30755689 PMC6372608

[4] Choi E Bahadori MT Schuetz A Stewart WF Sun J. Doctor AI: predicting clinical events via recurrent neural networks. *Proceedings of Machine Learning for Healthcare 2016 JMLR W&C Track 56*. Boston, MA: Proceedings of Machine Learning Research (2015). p. 1–12. 10.1002/aur.1474.Replication PMC5341604 28286600

[5] Choi E Bahadori MT Schuetz A Stewart WF Sun J. RETAIN: interpretable predictive model in healthcare using reverse time attention mechanism. In: *Proceedings of the 30th International Conference on Neural Information Processing Systems*. Red Hook, NY: Curran Associates, Inc. (2016). p. 3512–3520.

[6] Choi E Schuetz A Stewart WF Sun J. Using recurrent neural network models for early detection of heart failure onset. J Am Med Inform Assoc (2016) 292:344–50. 10.1093/jamia/ocw112 PMC5391725 27521897

[7] Sha Y Wang MD. Interpretable predictions of clinical outcomes with an attention- based recurrent neural network. In: *Proceedings of the 8th ACM International Conference on Bioinformatics, Computational Biology,, Health Informatics*. New York, NY: Association for Computing Machinery (2017). p. 233–240 10.1145/3107411.3107445 PMC7310714 32577628

[8] Lipton ZC Kale DC Elkan C Wetzell R. Learning to diagnose with LSTM recurrent neural networks. In: *4th International Conference on Learning Representations*. San Juan, Puerto Rico (2016).

[9] Lin L Xu B Wu W Richardson T Bernal EA. Medical time series classification with hierarchical attention-based temporal convolutional networks: a case study of myotonic dystrophy diagnosis. In: *CVPR Workshops*. New York, NY: Institute for Electrical and Electronics Engineers (2019). p. 83–86.

[10] Nguyen P Tran T Wickramasinghe N Venkatesh S. Deepr: a convolutional net for medical records (2016). p. 1–9. 10.1109/JBHI.2016.2633963 27913366

[11] Vaswani A Shazeer N Parmar N Uszkoreit J Jones L Gomez AN Attention is all you need. Adv Neural Inf Process Syst (2017) 30:5998–6008. 10.1017/S0952523813000308

[12] Devlin J Chang MW Lee K Toutanova K. BERT: pre-training of deep bidirectional transformers for language understanding [Preprint] (2018). Available at: arXiv:1811.03600v2.

[13] Zhou T Ma Z Wen Q Wang X Sun L Jin R. Fedformer: frequency enhanced decomposed transformer for long-term series forecasting. *CoRR* (2022). Available at: arXiv:abs/2201.12740.

[14] Li Y Rao S Roberto J Solares A Hassaine A Ramakrishnan R, BEHRT: transformer for electronic health records. Sci Rep (2020) 10:1–12. 10.1038/s41598-020-62922-y 32346050 PMC7189231

[15] Rasmy L. Med-BERT: pretrained contextualized embeddings on large- scale structured electronic health records for disease prediction. NPJ Digit Med (2021) 4:1–13. 10.1038/s41746-021-00455-y 34017034 PMC8137882

[16] Li Y Mamouei M Salimi-khorshidi G Rao S Hassaine A Canoy D Hi-BEHRT: hierarchical transformer-based model for accurate prediction of clinical events using multimodal longitudinal electronic health records. *arXiv* (2021). 10.1109/JBHI.2022.3224727 PMC7615082 36427286

[17] Song H Rajan D Thiagarajan JJ Spanias A. Attend, diagnose: clinical time series analysis using attention models. In: *Thirty-second AAAI Conference on Artificial Intelligence*. Red Hook, NY: Curran Associates, Inc. (2018).

[18] Beltagy I Peters ME Cohan A. Longformer: the long-document transformer. *arXiv* (2020).

[19] Meng C Trinh L Xu N Enouen J Liu Y. Interpretability, fairness evaluation of deep learning models on MIMIC-IV dataset. Sci Rep (2022) 12:1–28. 10.1038/s41598-022-11012-2 35504931 PMC9065125

[20] Dorogush AV Gulin A Gusev G Kazeev N Prokhorenkova LO Vorobev A. Fighting biases with dynamic boosting. *CoRR* (2017). Available at: abs/1706.09516.

[21] Chen T Guestrin C. XGBoost: a scalable tree boosting system. *CoRR* (2016). Available at: abs/1603.02754.

[22] Qin Y Song D Cheng H Cheng W Jiang G Cottrell GW. A dual-stage attention-based recurrent neural network for time series prediction. *International Joint Conference on Artificial Intelligence (IJCAI)*. Red Hook, NY: Curran Associates, Inc. (2017). p. 2627–2633.

[23] Sukhbaatar S Szlam A. End-to-end memory networks. In: *Advances in Neural Information Processing Systems*. Red Hook, NY: Curran Associates, Inc. (2015). p. 2440–2448.

[24] Han K Wang Y Chen H Chen X Guo J Liu Z A survey on vision transformer. IEEE Trans Pattern Anal Mach Intell (2022): 1–1. 10.1109/TPAMI.2022.3152247. https://ieeexplore.ieee.org/document/9716741 35180075

[25] Patrick M Campbell D Asano Y Misra I Metze F Feichtenhofer C Keeping your eye on the ball: trajectory attention in video transformers. In: Ranzato M, Beygelzimer A, Dauphin Y, Liang P, Vaughan JW, editors. *Advances in Neural Information Processing Systems*. Vol. 34. Curran Associates, Inc. (2021). p. 12493–12506.

[26] Huang K Altosaar J Ranganath R. ClinicalBERT: modeling clinical notes and predicting hospital readmission. *arXiv* (2019).

[27] Zhang H Lu AX Mcdermott M. HurtfulWords: quantifying biases in clinical contextual word embeddings. In: *Proceedings of the ACM Conference on Health, Inference, and Learning*. New York, NY: Association for Computing Machinery (2020). p. 110–120.

[28] Ren H Wang J Zhao WX. RAPT: pre-training of time-aware transformer for learning robust healthcare representation. In: *Proceedings of the 27th ACM SIGKDD Conference on Knowledge Discovery & Data Mining*. New York, NY: Association for Computing Machinery (2021). p. 3503–3511.

[29] Peng X Long G Shen T Wang S Jiang J. Sequential diagnosis prediction with transformer and ontological representation. *arXiv* (2021).

[30] Prakash P Chilukuri S Ranade N Viswanathan S. RareBERT: transformer architecture for rare disease patient identification using administrative claims. In: *The Thirty-Fifth AAAI Conference on Artificial Intelligence (AAAI-21) RareBERT*. Palo Alto, CA: AAAI Press (2021). p. 453–460.

[31] Meng Y Speier W Ong MK Arnold CW. Transformers using multimodal electronic health record data to predict depression. IEEE J Biomed Health Inform (2021) 25:3121–9. 10.1109/JBHI.2021.3063721 33661740 PMC8606118

[32] Choi E Xu Z Li Y Dusenberry MW Flores G Xue E Learning the graphical structure of electronic health records with graph convolutional transformer. In: *Proceedings of the AAAI Conference on Artificial Intelligence*. Palo Alto, CA: AAAI Press (2020). p. 606–613.

[33] Shang J Ma T Xiao C Sun J. Pre-training of graph augmented transformers for medication recommendation. *arXiv* (2019).

[34] Johnson AEW Stone DJ Celi LA Pollard TJ. The mimic code repository: enabling reproducibility in critical care research. J Am Med Inform Assoc (2018) 25:32–9. 10.1093/jamia/ocx084 29036464 PMC6381763

